# Steroidogenic Effects of Salinity Change on the Hypothalamus–Pituitary–Gonad (HPG) Axis of Male Chinese Sea Bass (*Lateolabrax maculatus*)

**DOI:** 10.3390/ijms231810905

**Published:** 2022-09-18

**Authors:** Zhenru Fang, Xujian Li, Yapeng Wang, Wei Lu, Juncheng Hou, Jie Cheng

**Affiliations:** 1 Key Laboratory of Marine Genetics and Breeding (Ocean University of China), Ministry of Education, 5 Yushan Road, Qingdao 266003, China; 2 Laboratory for Marine Fisheries Science and Food Production Processes, Pilot National Laboratory for Marine Science and Technology (Qingdao), 1 Wenhai Road, Qingdao 266237, China; 3 Key Laboratory of Tropical Aquatic Germplasm of Hainan Province, Sanya Oceanographic Institution, Ocean University of China, Sanya 572024, China

**Keywords:** hypothalamus–pituitary–gonad axis (HPG), salinity change, steroid hormone, sexual development, *Lateolabrax maculatus*

## Abstract

As lower vertebrates, teleost species could be affected by dynamic aquatic environments and may respond to environmental changes through the hypothalamus–pituitary–gonad (HPG) axis to ensure their normal growth and sexual development. Chinese sea bass (*Lateolabrax maculatus*), euryhaline marine teleosts, have an extraordinary ability to deal with a wide range of salinity changes, whereas the salinity decrease during their sex-maturation season may interfere with the HPG axis and affect their steroid hormone metabolism, resulting in abnormal reproductive functioning. To this end, in this study, 40 HPG axis genes in the *L. maculatus* genome were systematically characterized and their copy numbers, phylogenies, gene structures, and expression patterns were investigated, revealing the conservation of the HPG axis among teleost lineages. In addition, freshwater acclimation was carried out with maturing male *L. maculatus*, and their serum cortisol and 11-ketotestosterone (11-KT) levels were both increased significantly after the salinity change, while their testes were found to be partially degraded. After salinity reduction, the expression of genes involved in cortisol and 11-KT synthesis (*cyp17a*, *hsd3b1*, *cyp21a*, *cyp11c*, *hsd11b2*, and *hsd17b3*) showed generally upregulated expression in the head kidneys and testes, respectively. Moreover, *cyp11c* and *hsd11b2* were involved in the synthesis and metabolism of both cortisol and 11-KT, and after salinity change their putative interaction may contribute to steroid hormone homeostasis. Our results proved the effects of salinity change on the HPG axis and steroidogenic pathway in *L. maculatus* and revealed the gene interactions involved in the regulation of steroid hormone levels. The coordinated interaction of steroidogenic genes provides comprehensive insights into steroidogenic pathway regulation, as well as sexual development, in teleost species.

## 1. Introduction

Environmental changes to aquatic ecosystems, such as variation in water temperature, salinity, pH, and oxygen content, could influence the physiology, reproduction, and behavior of teleost species, including the processes of steroidogenesis and gametogenesis in the gonads [1,2,3]. In vertebrates, steroidogenesis occurs mainly in tissues, such as the gonads, the inter-renal gland (the head kidney in teleosts), and the brain, which, in general, is controlled by the hypothalamus–pituitary–inter-renal (HPI) and the hypothalamus–pituitary–gonad (HPG) axes, and has a considerable impact on the regulation of the complex steroidogenesis process and affects reproduction [4,5].

The HPG axis plays an essential role in teleost growth and reproduction [6]. The HPG axis is initiated by the secretion of gonadotropin-releasing hormone (GnRH) from neuroendocrine cells in the hypothalamus, which acts on the pituitary to promote follicle-stimulating-hormone (FSH) and luteinizing-hormone (LH) release and activate the FSH and LH receptors in the gonads, ultimately leading to the synthesis of steroid hormones [6,7], which are key factors in sex determination and differentiation as well as germ cell development [8,9]. Steroidogenesis is further triggered by steroidogenic acute regulatory protein (Star), mobilizing cholesterol from the outer to the inner mitochondrial membrane [10,11]. The following process employs diverse enzymes, such as cytochrome P450s and hydroxysteroid dehydrogenases, which participate in the synthesis of C18 estradiol (E2), C19 testosterone (T), and C21 cortisol [4,5]. *Cytochrome P450* (*cyp*) is a multigene family involved in catalyzing the production of sex steroids [12]. For example, Cyp11a is the sole enzyme that catalyzes the generation of pregnenolone, which process is the initiation step in steroid hormone biosynthesis [13,14]. Cyp11b/c can promote the production of cortisol and 11β-OH-testosterone, which has a great impact on male testicular development and spermatogenesis [5,15]. Cyp17a1 and Cyp17a2 are two isoforms of steroid 17α-hydroxylase/17,20-lyase, with Cyp17a1 possessing both 17α-hydroxylase and 17,20-lyase activities, while Cyp17a2 has only 17α-hydroxylase activity [16,17]. Aromatase (Cyp19a1) is responsible for the transformation of androstenedione into estrone and testosterone into estradiol, in which the regulation of *Cyp19a1* is crucial for ovarian and testicular differentiation [18,19]. In addition, *hydroxysteroid dehydrogenase*s (*hsd*s) are also involved in the regulation of steroid-hormone biosynthesis [20]. For instance, 3 beta-hydroxysteroid dehydrogenase (Hsd3b) is important in the production of progesterone and testosterone [21]. Corticosteroid 11-beta-dehydrogenase isozyme 2 (Hsd11b2) is involved in the conversion of cortisol to cortisone, as well as 11-oxygenated androgen synthesis [5,22]. Moreover, various forms of Hsd17bs have been reported in teleosts, which participate in the production of essential sex steroids, such as T and E2 [23,24,25].

Salinity is one of the most important environmental factors that greatly affects the survival, reproduction, growth, development, and physiological functions of teleosts [26,27]. Recent research has suggested that cortisol, mostly associated with stress response via the HPI axis, may act as a key factor linking environmental stimuli to physiological responses during sexual development and reproduction through its cross-talk with the HPG axis, initiating a shift in steroidogenesis from estrogens to androgens [1,3,27]. Therefore, changes in salinity could have major effects on teleost sexual development, such as spermatogenesis and testicular homeostasis, as well as the proliferation and apoptosis of gonadal cells [2,28]. Chinese sea bass (*Lateolabrax maculatus*) is one of the most popular aquaculture fish species in China due to its high nutritive value and pleasant taste. Juvenile *L. maculatus* normally grow in estuarine areas, and when sexually mature they move to waters with relatively high salinity. In addition, *L. maculatus* is known for its ability to adapt to a wide range of salinity environments, from freshwater (0‰, FW) to seawater (30‰, SW), as well as alkaline water [29,30]. However, salinity changes, such as acclimation to freshwater, were reported to affect gonadal development and differentiation in teleost species [3,31], and only a limited number of studies have focused on the steroidogenic effect of salinity change on the HPG axis of *L. maculatus*. Therefore, in this study, the HPG axis genes in *L. maculatus* were characterized and their evolution, expression, and coordinated interaction in response to salinity change were investigated. These results will provide a comprehensive understanding of the relationship between the regulation of the steroidogenic pathway and sexual development in teleost species under diverse environmental stimuli.

## 2. Results and Discussion

### 2.1. Genomic Landscape of HPG Axis Genes in L. maculatus

**Figure 1:**
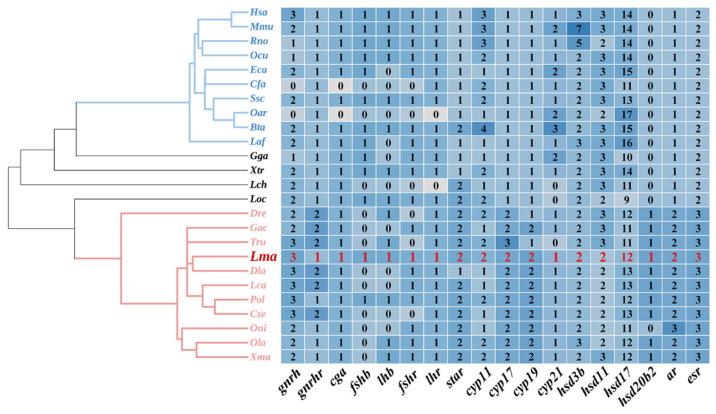
Copy numbers of HPG axis genes in selected vertebrate genomes presented in a heatmap, along with their phylogeny. Copy number comparisons were made among the HPG axis genes in 10 mammals, chicken, frog, spotted gar, coelacanth, and 11 teleosts. In the phylogenetic tree, blue lines represent mammalian lineages and pink lines represent teleost lineages. Abbreviations: human (*Hsa*), monkey (*Mmu*), Norwegian rat (*Rno*), rabbit (*Ocu*), horse (*Eca*), dog (*Cfa*), pig (*Ssc*), sheep (*Oar*), cattle (*Bta*), African savanna elephant (*Laf*), chicken (*Gga*), tropical clawed frog (*Xtr*), coelacanth (*Lch*), spotted gar (*Loc*), zebrafish (*Dre*), stickleback (*Gac*), pufferfish (*Tru*), Chinese sea bass (*Lma*), European sea bass (*Dla*), Asian sea bass (*Lca*), Japanese flounder (*Pol*), Chinese tongue sole (*Cse*), tilapia (*Oni*), Japanese medaka (*Ola*), platyfish (*Xma*).

A total of 40 HPG axis genes were identified in the *L. maculatus* genome, including hypothalamus- and pituitary-distributed *gnrh*, *gnrhr*, *cga*, *lhb*, *fshb*, *lhr*, and *fshr* and gonad-distributed *star*, *cyp11*, *cyp17*, *cyp19*, *cyp21*, *hsd3b*, *hsd11*, *hsd17*, *hsd20*, *ar*, and *esr* (Figure 1). The copy numbers of most HPG axis genes were conserved among the selected tetrapod and teleost lineages, with some exceptions. For example, *star*, *cyp17a*, *cyp19a1*, *hsd17b12*, *ar*, and *esr2* were single-copy genes in tetrapods, whereas duplicated copies of these genes were found in teleosts (Figure 1 and Table S1), among which *cyp19a1a/b*, *hsd17b12a/b*, *ara/b*, and *esr2a/b* were reported as having originated from a teleost-specific third-round genome-duplication (TSGD) event during vertebrate evolution [9,32]. Therefore, these duplicated genes might exhibit functional differentiation [9]. For instance, in zebrafish (*Danio rerio*) and other teleosts, *cyp19a1a* functions mainly in the gonads, catalyzing the production of estrogen, while *cyp19a1b* functions mainly in the brain [33,34]. Regarding *star* and *star2*, *star* transports cholesterol from the outer to the inner mitochondrial membrane, whereas *star2* has a role in testicular development, spermatogenesis, and spermiation by regulating androgen production in tilapia (*Oreochromis niloticus*) [35,36]. Furthermore, the *hsd3b*, *hsd11b*, *hsd17b*, and *hsd20b* genes are pivotal members of the *hsd* gene family. In total, 17 *hsd* genes were identified in the *L. maculatus* genome, which were generally conserved between tetrapods and teleosts, with only *hsd20b* present in teleosts and not in tetrapods (Figure 1) [37]. Two or three *hsd3b* genes were identified in most selected vertebrates as the conserved *hsd3b1* and *hsd3b7*, while *hsd3b2*, *-3*, *-4*, *-5*, and *-6* were only found in mice (*Mus Musculus*) (Table S1). In addition, *hsd17b* was the largest *hsd* family, with 12 *hsd17b* members being found in *L. maculatus* (Figure 1).

### 2.2. Phylogeny of HPG Axis Genes among Selected Vertebrates

**Figure 2:**
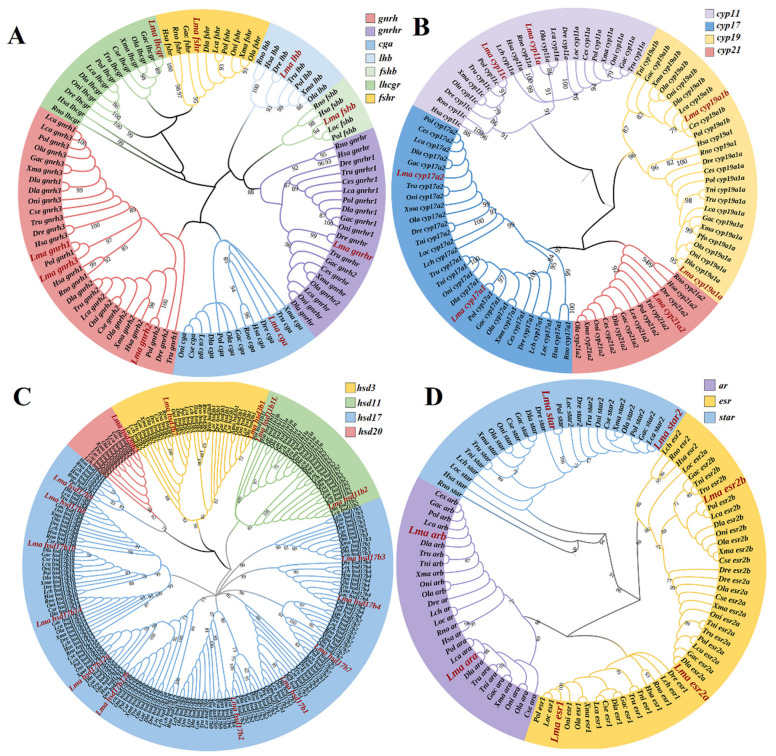
Phylogeny of the HPG axis genes among vertebrates. (**A**) Genes mainly present in the hypothalamus and pituitary (*gnrh*, *gnrhr*, *cga*, *lhb*, *fshb*, *lhr,* and *fshr*). (**B**) The *cyp* gene family. (**C**) The *hsd* gene family. (**D**) The *star*, *ar*, and *esr* genes. Colored ranges represent different gene classes. The *L. maculatus* HPG axis genes are shown in red font, enlarged italics, and bold.

To reveal the evolutionary relationships of HPG axis genes in *L. maculatus*, their phylogeny was investigated, and the expected clades were supported by strong bootstrap values (Figure 2). Specifically, the hypothalamus- and pituitary-distributed genes *gnrh*, *gnrhr*, *cga*, *lhb*, *fshb*, *lhr*, and *fshr* were well-clustered into their corresponding clades with other teleost lineages (Figure 2A). In addition, seven *cyp* genes were identified in the *L. maculatus* genome, and the vertebrate *cyp* genes were clustered into four clades corresponding to the *cyp11*, *cyp17a*, *cyp19a*, and *cyp21a* families (Figure 2B). The *cyp17a* clade was further divided into two branches, with the teleost *cyp17a1*s clustered with tetrapod homologs, while teleost *cyp17a2*s were further clustered together (Figure 2B). The *cyp19a1* clade was also grouped into two branches, which corresponded to *cyp19a1* of tetrapods and *cyp19a1a/cyp19a1b* of teleosts (Figure 2B), confirming their TSGD origin in teleost lineages.

Moreover, most *hsd* genes were well-clustered with their homologs, with *hsd17b12a* and *hsd17b12b* clustered into two branches in teleosts and further clustered with tetrapod *hsd17b12*s, suggesting their TSGD origin in teleosts from the ancestor *hsd17b12* (Figure 2C). In addition, a single copy of *star* was present in tetrapods, while two copies were identified in most teleost species as *star* and *star2* (Figure 1). With regard to *esr*s, *esr1* was conserved as a single copy among selected vertebrates, whereas there were two *esr2*s, *esr2a* and *esr2b*, found in most teleost species (Table S1). This was also the case for *ar* genes, with *ara* and *arb* found to be present in the teleost lineages (Table S1). Moreover, *star* and *star2* were split into two clades, similar to the findings of Yang et al. [9]. *Esr*s were also clustered into two clades, *esr1* and *esr2*, with *esr2* further divided into two branches (Figure 2D): *esr2* in tetrapods and *esr2a* and *esr2b* in teleosts.

### 2.3. Conserved Sequence Structures of HPG Axis Genes among Teleost Species

**Figure 3:**
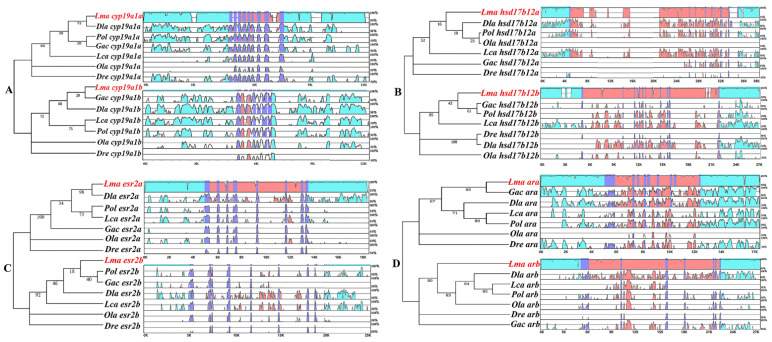
Conserved sequence structures of HPG axis genes in teleost lineages, as determined by mVISTA analysis. (**A**) *cyp19a1a/cyp19a1b*. (**B**) *hsd17b12a/hsd17b12b*. (**C**) *esr2a*/*esr2b*. (**D**) *ara/arb*. Blue coloring represents intergenic regions; purple coloring represents exon regions; orange coloring represents intron regions. The similarities in gene structure among *L. maculatus* and selected teleosts that were higher than 50% are displayed in color. The genes of the *L. maculatus* HPG axis are shown in red font, enlarged italics, and bold.

For the putatively duplicated steroidogenic genes, a previous study indicated their varied evolutionary patterns in teleost lineages [9]. For instance, through molecular evolutionary analysis, *star2*, *cyp17a2*, and *cyp19a1b* showed faster evolutionary rates (ω_1_) than their paralogs (ω_0_) [9], indicating their possible functional divergence independent of the TSGD. To further investigate the structural conservation and variability of the putatively duplicated genes in the HPG axis of teleosts, an mVISTA analysis was performed for the selected seven pairs of genes in teleost lineages, including the gene sequences and the up- and downstream neighboring regions of *cyp19a1a/cyp19a1b*, *hsd17b12a/hsd17b12b*, *esr2a*/*esr2b*, and *ara/arb* (Figure 3) as well as of *star/star2*, *cyp11a/cyp11c*, and *cyp17a1/cyp17a2* (Figure S1). According to the results, the coding exon regions (purple) were highly conserved among the genes in teleosts, except for *star* and *cyp17a1*, while the intron regions (orange) showed strong diversity among teleost species (Figure 3 and Figure S1). Moreover, several conserved non-coding regions (blue), mostly related to the putative transcription-factor binding sites (TFBSs) in the 5′ upstream regions, were identified in *cyp19a1a*, *cyp19a1b*, *ara*, *esr2a*, *cyp11a*, and *cyp11c* (Figure 3 and Figure S1), indicating the general functional importance of these conserved regions in the gonad cells of teleost lineages, which were also found to be independent of gene duplication.

### 2.4. Expression of HPG Axis Genes in L. maculatus

Gene-expression profiling facilitates understanding of the function and evolution of HPG axis genes. Transcriptome analyses were performed for seven adult tissues of *L. maculatus* to investigate the expression profiles of 40 HPG axis genes. As a result, steroidogenesis during sexual development in teleosts was found to be essentially regulated by the differential expression of several steroidogenic enzymes, most of which presented conserved expression patterns in *L. maculatus* (Figure S2). For example, in *L. maculatus*, *gnrh*, *gnrhr*, *cga*, *fshb*, *lhb*, and *fshr* were intensively distributed in the brain, *cyp19a1a*, *cyp11a*, and *hsd17b12* were most abundantly observed in the ovaries, while *cyp11c*, *cyp17a*, *hsd3b1*, *hsd11b2*, *hsd17b7*, and *hsd17b14*, as well as several sex hormone receptors (*arb*, *esr1*, and *esr2a/b*), were intensively expressed in the testes, which patterns were similar to those observed in other teleost species [9] and suggested the functional conservation of the *L. maculatus* HPG axis.

### 2.5. Effects of Salinity Change on HPG Axis Genes and the Sexual Development of Male L. maculatus

Steroid biosynthesis in teleosts is mainly controlled by the HPI and HPG axes, including a series of enzyme reactions from cholesterol to produce cortisol, estrogen (estradiol, E2), and androgen (11-ketotestosterone, 11-KT) [4]. In order to illustrate the steroidogenic effects of salinity change on HPG axis gene expression, steroid hormone biosynthesis, and testis development, a 15-day freshwater (FW) acclimation experiment was conducted with maturing 3-year-old male *L. maculatus*.

**Figure 4:**
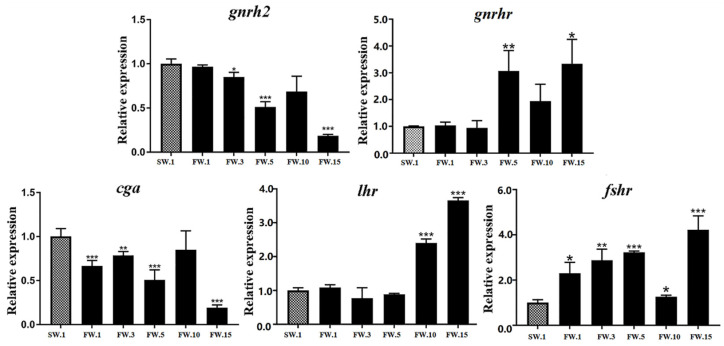
Expression of hypothalamus- and pituitary-related HPG axis genes in the brains of male *L. maculatus* in response to salinity change. * represents *p* < 0.05; ** represents *p* < 0.01; *** represents *p* < 0.001.

Firstly, the brain directs gonadal development through GnRH, the main regulator of reproduction in vertebrates in the hypothalamus, which modulates the release of both gonadotropins, FSH and LH, from the pituitary [6,38]. After the 15-day FW acclimation, expression levels of *gnrh2* and *cga* were significantly decreased in the *L. maculatus* brain, while their receptors *gnrhr*, *lhr*, and *fshr* generally showed upregulated expression, especially from the 5th to the 15th day (Figure 4 and Table S2). These results were similar to those of a previous study [31], which suggested that the mRNA levels of GnRH, FSH, and LH subunits may not necessarily reflect changes in circulating hormone levels in *L. maculatus* under salinity change.

**Figure 5:**
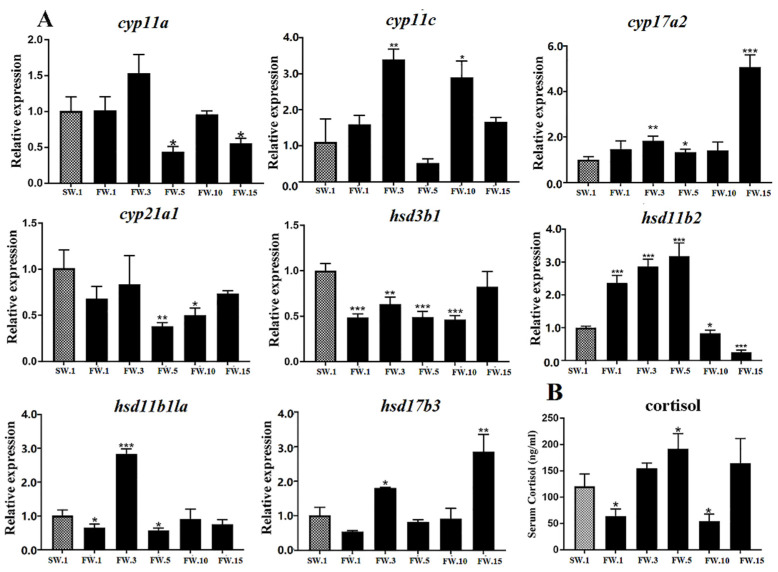
Cortisol synthesis regulation in the head kidney of male *L. maculatus* in response to salinity change. (**A**) Expression of candidate steroidogenic genes in the head kidney of male *L. maculatus*. (**B**) Serum cortisol levels in male *L. maculatus* after the freshwater acclimation. * represents *p* < 0.05; ** represents *p* < 0.01; *** represents *p* < 0.001.

It is well known that the HPI axis could transduce environmental stimuli to physiological responses and finally promote the synthesis and release of cortisol from the inter-renal gland, which comprises specialized cells in the head kidney of teleost fish and is functionally homologous to the adrenal gland in mammals [4]. Under FW acclimation, dynamic gene regulation patterns were observed in the head kidney of *L. maculatus* (Figure 5A). For example, in the head kidney, cholesterol was transformed into progesterone under the action of *cyp11a* and *hsd3b1* and further into 17α-OH-progesterone under the action of *cyp17a2* [5]. According to qRT-PCR, both *cyp11a* and *hsd3b1* represented downregulated expression after the FW acclimation, whereas *cyp17a2*, but not *cyp17a1*, showed upregulation, especially at the 15th day (Figure 5A and Table S2). A previous study proved that *cyp17a1* participated in the generation of estradiol in the ovary, while the generation of C21 steroids, such as cortisol, in the head kidney was found to be associated with *Cyp17a2* [17]. Then, 17α-OH-progesterone was transformed into cortisol under the interaction of *cyp21a1* and *cyp11c*. According to qRT-PCR, *cyp21a1* and *cyp11c* showed different expression patterns after salinity reduction (Figure 5A). The expression of *cyp11c* gradually increased at the 1st and 3rd days and reduced at the 5th day, then increased significantly at the 10th and 15th days (Figure 5A and Table S2). However, the upstream *cyp21a1* was downregulated throughout the experiment, which may indicate its having a novel function in cortisol biosynthesis (Figure 5A). Moreover, cortisol is the main glucocorticoid directly associated with stress in teleosts [39]. The serum cortisol levels in the FW group changed significantly during acclimation; they declined at the 1st day and increased from the 3rd to the 5th days, then decreased sharply at the 10th day and increased to levels comparable to those of the SW group at the 15th day (Figure 5B and Table S3). The increased expression of *cyp11c* may have led to the upregulation of cortisol levels in *L. maculatus* under salinity change, similar to the dynamic patterns observed for peaks in cortisol release and then recovery to baseline levels after stresses in other teleosts [40].

**Figure 6:**
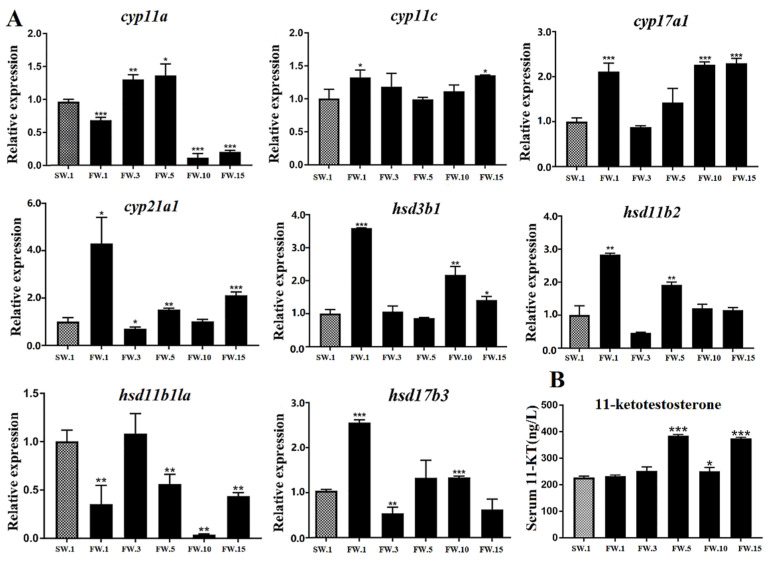
Synthesis regulation of 11-KT in the testes of male *L. maculatus* in response to salinity change. (**A**) Expression of candidate steroidogenic genes in the testes of male *L. maculatus*. (**B**) Serum 11-KT levels in male *L. maculatus* after the freshwater acclimation. * represents *p* < 0.05; ** represents *p* < 0.01; *** represents *p* < 0.001.

In gonad tissues, where sex steroid hormones are produced in leydig cells, cholesterol was transformed into progesterone under the action of *cyp11a* and *hsd3b1* and further into androstenedione under the action of *cyp17a1*, but not *cyp17a2* [5]. According to qRT-PCR, *cyp17a1* and *hsd3b1* showed similar expression patterns of significant upregulation at the 1st day, followed by decreases to levels similar to those of the control group at the 3rd and 5th days, then significant increases at the 10th and 15th days of salinity reduction (Figure 6A and Table S2). Furthermore, androstenedione was transferred into testosterone and 11-KT under the coregulation of *hsd17b3*, *cyp11c*, and *hsd11b2*. According to qRT-PCR, *hsd11b2* and *hsd17b3* responded to salinity changes according to similar patterns, with the expression of both genes significantly upregulated at the 1st day, reduced at the 3rd day, and then increased to higher levels than those of the control group at the 5th and 10th days (Figure 6A and Table S2). Most of the genes above showed similar expression patterns in response to salinity change, that is, they rose first, then fell, then rose again, similar to serum cortisol levels (Figure 5B). In addition, 11-KT is the steroid hormone produced especially in teleost gonads and is the most important androgen in teleost species. Serum 11-KT levels in *L. maculatus* gradually increased from the 5th day to the 15th day (Figure 6B and Table S3). This change was similar to those of genes involved in testosterone production, such as *cyp17a1*, *hsd17b3*, *hsd11b2*, and *hsd3b1*. Interestingly, *cyp11c* is involved in the production of both cortisol and 11-KT, and its expression level changed differently to those of other genes, with a generally higher expression level than the control group observed throughout the experiment (Figure 6A). Studies have shown that, with environmental stresses, cortisol levels in teleost fish could increase rapidly, then decrease, and finally recover to a slightly higher level than those of initial control groups (Figure 5B) [31,40]. From the 5th day of salinity reduction, *L. maculatus* may have adapted to the freshwater environment, and the 11-KT level after salinity change was similar to the level of cortisol, which is consistent with the related steroidogenic gene expression patterns.

**Figure 7:**
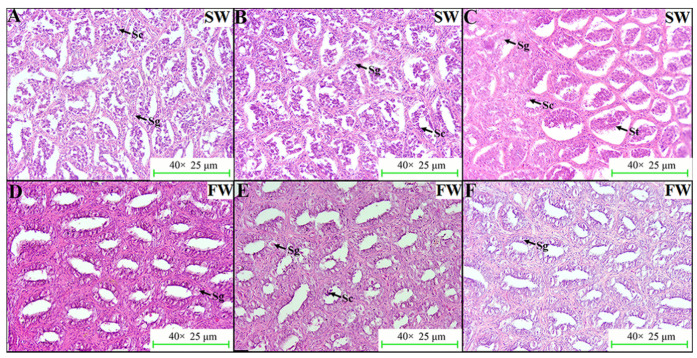
Effects of salinity change on *L. maculatus* testes with H&E staining. (**A**–**C**) Testes slides from three specimens of the seawater (SW) group. (**D**–**F**) Testes slides from three specimens of the freshwater (FW) group. The pictures are shown as 40× views. Sg: spermatogonia; Sc: spermatocyte; St: spermatid.

In addition, the histological changes to *L. maculatus* testes under FW acclimation were also investigated. As a result, late-spermatogenesis-stage testes were observed in the SW group at day 0, and seminiferous tubules filled with spermatocytes and spermatids could be obviously identified (Figure 7A–C). However, the maturity status of *L. maculatus* testes was reduced after acclimation in FW for 15 days when compared to the SW group, in which spermatogonia, but few spermatocytes and spermatids could be identified, and spermatogonia were the dominant germ cells in *L. maculatus* testes (Figure 7D–F).

### 2.6. Putative Interaction between the HPG and HPI Axes in Male L. maculatus under Salinity Change

**Figure 8:**
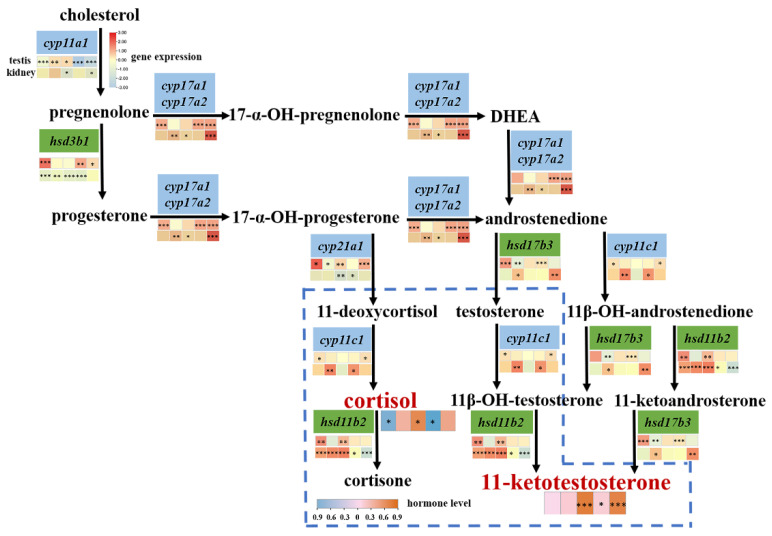
Putative steroid hormone biosynthesis pathway in *L. maculatus*, with expression responses to salinity change. The interactive cross-talk between cortisol and 11-KT biosynthesis pathways is indicated with the dashed-line frame. The heatmaps under each gene show their expression profiles after FW acclimation, while the heatmaps under cortisol and 11-KT show steroid levels. * represents *p* < 0.05; ** represents *p* < 0.01; *** represents *p* < 0.001.

The biosynthesis of 11-KT and cortisol from cholesterol metabolism involves a cascade reaction of multiple enzymes, the interactions of which could jointly regulate the development of fish reproductive systems [39]. For example, the process leading from cholesterol to 11-KT mainly involves *hsd3b1*, *cyp17a1*, *cyp11c*, *hsd11b2*, and *hsd17b3*, while the process leading from cholesterol to cortisol and further to cortisone mainly involves *hsd3b1*, *cyp17a2*, *cyp21a1*, *cyp11c*, and *hsd11b2* (Figure 8). The expression patterns of the above genes in response to salinity change were also consistent with the Pearson’s correlation coefficients (PCCs) (Figure S3). For example, the expression of *cyp21a1*, *hsd3b1*, *hsd11b2*, and *hsd17b3* were similar and suggested strong positive correlations (PCC > 0.7, *p* < 0.05), while *cyp11c* showed moderate correlation with *cyp17a2* and *cyp21a1* (PCC > 0.5, *p* < 0.05) (Figure S3 and Table S4). Both *cyp11c* and *hsd11b2* could participate in the metabolism of cortisol and 11-KT. Therefore, it is speculated that *cyp11c*, in response to salinity change, may first affect cortisol biosynthesis and then affect 11-KT production (Figure 8). Moreover, as mentioned above, an increase in cortisol level could induce high androgen synthesis by upregulation of *hsd11b2*, which encodes the enzyme involved in the conversion of cortisol to inactive cortisone and the synthesis of 11-KT (Figure 8) [38]. Therefore, the upregulated *hsd11b2* in *L. maculatus* testes was found to be strongly correlated with other genes for 11-KT production (Figure 6A) and may also contribute to steroid homeostasis by reducing cortisol levels. This role was similar to that ascribed to *hsd11b2* with the suggestion that it protects testicular tissues from circulating cortisol in addition to its role in 11-KT production, while the other form, *hsd11b1*, was found not to be well characterized in teleosts [5] and may not contribute to the cross-talk between cortisol and androgen pathways.

**Figure 9:**
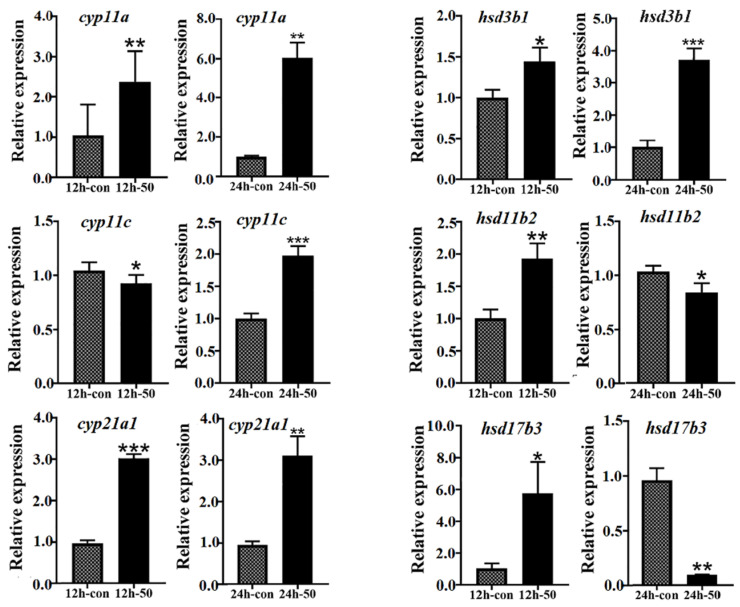
Expression of steroidogenic genes in response to cortisol in vitro stimuli in testes of *L. maculatus*. * represents *p* < 0.05; ** represents *p* < 0.01; *** represents *p* < 0.001.

Moreover, as an important androgen in teleosts, 11-KT plays an essential role in regulating fish reproduction and spermatogenesis, and its expression level is affected by cortisol hormone level [39]. It has been shown that the interaction between *cyp11c* and *hsd11b2* can jointly affect testosterone/11-KT and cortisol levels in teleosts [5,39]. For example, it has been shown that cortisol induces spermatogonial mitosis by increasing the production of 11-KT [39,41], and high-dose cortisol could compete with *cyp11c* and *hsd11b* for common carp (*Cyprinus carpio*) steroidogenesis [39]. Moreover, the dual role of enzymes involved in cortisol and androgen biosynthesis has also been demonstrated in rainbow trout (*Oncorhynchus mykiss*) [39,42], and low-dose cortisol administration has been shown to induce spermatogenesis and enhance spermatogonial proliferation by in vitro synthesis of 11-KT in spermatogenic explants of Japanese eel (*Anguilla japonica*) [39,43]. Cortisol produced from the head kidney may act via glucocorticoid receptors (GRs) and critically regulate the promoter functions of *cyp11c* or *hsd11b2* [39,41]. For example, cortisol could induce GRs to activate the promoter activity of *hsd11b2* in the testes of pejerrey (*Odontesthes bonariensis*) or the European eel (*Anguilla Anguilla*) in male-favorable temperatures [41]. Therefore, cortisol in vitro stimulation of testis tissues of *L. maculatus* showed that most of the steroidogenic genes in the testes were upregulated, with some exceptions (Figure 9). Interestingly, *cyp11c* and *hsd11b2* showed opposite regulation patterns, with *cyp11c* being downregulated but *hsd11b2* upregulated after 12 h of cortisol stimuli, while *cyp11c* was upregulated but *hsd11b2* downregulated after 24 h of cortisol culture (Figure 9). The explanation for this result is probably that at the beginning of the stimulation with a high cortisol dose, *cyp11c* was inhibited and limited cortisol production, while *hsd11b2* was activated and catalyzed overproduced cortisol into cortisone, whereas, as the cortisol was consumed after 24 h, *cyp11c* was reactivated to produce 11-KT, while *hsd11b2* was inhibited to keep a stable cortisol level. Even cortisol effects are dose- and species-specific [39], our results suggesting a putative correlated interaction between the key enzymes *cyp11c* and *hsd11b2*. The precise regulatory mechanism of cortisol on steroidogenic genes in *L. maculatus*, especially *cyp11c* and *hsd11b2*, warrants further investigation to illustrate the interactive cross-talk between cortisol and androgen pathways in steroid homeostasis and sexual development of *L. maculatus* in response to diverse environmental changes.

## 3. Materials and Methods

### 3.1. Identification and Annotation of HPG Axis Genes in L. maculatus

The coding sequence (CDSs) and amino acid (AA) sequences of HPG axis genes were identified from the *L. maculatus* genome (PRJNA408177) by BLAST search using homologous gene sequences from 24 selected vertebrate species, including 10 mammals, human (*homo sapiens*), mouse (*Mus musculus*), Norway rat (*Rattus norvegicus*), rabbit (*Oryctolagus cuniculus*), horse (*Equus caballus*), dog (*Canis lupus familiaris*), pig (*Sus scrofa*), sheep (*Ovis aries*), cattle (*Bos taurus*), and African savanna elephant (*Loxodonta africana*); chicken (*Gallus gallus*); tropical clawed frog (*Xenopus tropicalis*); coelacanth (*Latimeria chalumnae*); spotted gar (*Lepisosteus oculatus*); and 10 teleosts, zebrafish (*Danio rerio*), stickleback (*Gasterosteus aculeatus*), takifugu (*Takifugu rubripes*), Asian sea bass (*Lates calcarifer*), European sea bass (*Dicentrarchus labrax*), Japanese flounder (*Paralichthys olhaceus*), Chinese tongue sole (*Cynoglossus semilaevis*), tilapia (*Oreochromis niloticus*), Japanese Medaka (*Oryzias latipes*), and platyfish (*Xiphophorus maculatus*), from the Ensemble (http://asia.ensembl.org, accessed on 1 February 2022) and NCBI databses (http://www.ncbi.nlm.nih.gov, accessed on 1 February 2022). The copy numbers of HPG axis genes were illustrated in heatmaps using TBtools v1.09 [44].

### 3.2. Phylogenetic and Conserved Motif Analysis

Phylogenetic analysis was conducted using predicted CDSs of *L. maculatus* HPG axis genes together with the CDSs from 10 mammals, chicken, frog, coelacanth, spotted gar, and the 10 teleost species mentioned above. The multiple sequence alignments were performed with MUSCLE [45] using MEGA 7.0 [46], and the phylogenetic tree was constructed using IQtree [47] based on the maximum-likelihood (ML) method and the Jones–Taylor–Thornton (JTT) model with a bootstrap value of 1000. The final visualization was generated using iTOL v1.09 [48].

### 3.3. Conserved Sequence Analysis by mVista

Sequence conservation of the putatively duplicated HPG axis genes was further investigated among selected teleost species, including *L. maculatus*, *L. calcarifer*, *D. labrax*, *D. rerio*, *G. aculeatus*, *p. olivaceus*, and *O. latipes*, by mVISTA (https://genome.lbl.gov/vista/index.shtml, accessed on 1 April 2022), with sequences of the candidate genes covering the CDSs and the 5000 bp upstream and downstream intergenic regions. Gene sequences of other teleosts were compared with those of *L. maculatus* with the selected mode of LAGAN. When the similarity between sequences was higher than 50%, a colored peak was displayed, with blue representing intergenic regions, orange representing intron regions, and purple representing exon regions.

### 3.4. Expression of HPG Axis Genes in L. maculatus Tissues

To investigate the expression profiles of HPG axis genes in different *L. maculatus* tissues, transcriptome data for seven adult tissues were obtained from NCBI (brain-SRR7528887, stomach-SRR7528884, spleen-SRR7528888, liver-SRR7528886, gill-SRR7528883, testis-SRR7528885, and ovary-SRR2937376). Through the Hisat and StringTie pipeline [49], the fragments per kilobase of exon per million mapped reads (FPKMs) of the HPG axis genes were obtained. The heatmap was generated with log_2_(FPKM+1) via TBtools [44].

### 3.5. Salinity-Change Experiment on Maturing Male L. maculatus

To explore the effects of salinity change on the sexual development of male *L. maculatus*, a 15-day freshwater acclimation experiment was conducted. Three-year-old maturing *L. maculatus* (body length: 42.0 ± 1.0 cm; body weight: 728.79 ± 61.0 g) from seawater (30‰ and 18.0 ± 2.0 °C) were obtained from the fishery market in Qingdao, China. After seven days of acclimation, they were randomly divided into a control group (seawater, SW) and a treatment group (freshwater, FW), with a salinity reduction of 5‰ per 12 h from SW to FW. Then, the FW group was sampled on the 1st, 3rd, 5th, 10th, and 15th days, with three males sampled at each time point. A photoperiod of 10 light hours and 14 dark hours was used. After the acclimation, brain, head kidney, and gonad tissues were sampled with liquid nitrogen and stored at −80 °C for subsequent RNA extraction. Testis samples were also fixed with 4% paraformaldehyde (PFA) for 24 h, then routinely processed and embedded in paraffin for immunohistochemical studies. Blood was precipitated at 4 °C overnight and centrifuged at 4 °C (1000 RPM, 10 min), and was then stored at −20 °C for subsequent usage to measure serum hormone levels.

### 3.6. RNA Extraction and qRT-PCR of HPG Axis Genes in L. maculatus

Total RNA was extracted using TRIzol^®^ reagent. The concentration and integrity of RNA were examined by electrophoresis. Genomic DNA was eliminated and cDNA was synthetized using the PrimeScript™ RT reagent Kit with gDNA Eraser. The cDNA samples were subsequently used as templates for qRT-PCR experiments with HPG axis genes. All gene-specific primers were designed using the IDT program (Table S5). *ACTB* and *18S ribosomal RNA* [50] were used as internal controls, and the samples were repeated in triplicate technical repeats. Meanwhile, three male samples from each sampling time point were pooled as biological replicates. The total volume of the reaction was 20 μL, containing 2 μL template cDNA, 0.4 μL each forward/reverse primers, 10 μL SYBR^®^FAST qPCR Master Mix (2×), and 7.2 μL of nuclease-free water. The qRT-PCR process was as follows: 95 °C for 30 s, followed by 40 cycles at 95 °C for 5 s and 58 °C for 30 s. Relative expression levels were calculated by the 2^−ΔΔCt^ method.

### 3.7. Quantification of Serum Steroid Hormones in L. maculatus

Enzyme linked immunosorbent assays (ELISAs) were conducted to measure the serum cortisol and 11-KT levels using a Cortisol Assay Kit and an 11-keto Testosterone Kit (Nanjing Jiancheng Bioengineering Institute), following the manufacturers’ instructions. Before the measurements, all reagents were put at ambient temperature for at least 30 min for stabilization. The kits have three parts: (1) a zero well, without any sample but with chromogen solution A, B and stop solution for zero-setting; (2) a standard well (add 50 μL diluted standard to each well, add 50 μL standard/sample dilution to the zero well, and add 50 μL Biotinylated Ab to the working solution); and (3) a sample well (add 50 μL sample, then add 50 μL Biotinylated Ab working solution). All samples required three technical repetitions. After the well plating, shake the plate gently after sealing with the closure plate membrane, incubate for 30 min at 37 °C, then add wash solution to each well, discard after stabilizing for 30 s, repeat the action 5 times, and add 50 μL HRP working solution to the zero, standard, and sample wells, then incubate for 30 min at 37 °C. The second washing step is then conducted. Finally, add 50 μL chromogenic solution A and B to each well, incubate for 10 min at 37 °C away from light, then add 50 μL stop solution to each well to stop the reaction (the blue color changes to yellow immediately). Make the zero setting with an empty well, and measure the optical density (OD) of each well under a 450 nm wavelength with a Biotek Synergy^TM^ H1 microplate reader.

### 3.8. Histological Examination of L. maculatus Testes

To observe the testis status of *L. maculatus* after salinity change, testis sections were processed. The PFA-fixed testes were treated with a methanol gradient (30%, 50%, 70%, 80%, 90%, 95%, 100%). After anhydrous ethanol dehydration, xylene infiltration, and making the tissues transparent, the tissues were soaked in a paraffin environment at 60–65 °C for more than 3 h. The tissues were then sliced with a microtome, with a thickness of not more than 5 μM. The slices were incubated for 12 h at 37 °C to dry the water and then stained according to the following procedure: apply xylene infiltration twice, a mixture of xylene and ethanol (1:1), then anhydrous ethanol twice, 5 min each time for each step; then apply a 95%, 80%, 70%, 50%, 30% anhydrous ethanol gradient, distilled water twice, 2 min each time for each step; hematoxylin staining for 15 s; then reverse the above experimental steps; perform eosin staining for 6 s after a 95% ethanol gradient; apply a neutral gum seal; and observe with a microscope (SMZ-B6).

### 3.9. Correlation of HPG Axis Genes in L. maculatus with Serum Hormones

OmicShare (https://www.omicshare.com/, accessed on 16 June 2022) was employed for the Pearson’s correlation coefficient (PCC) analysis between the steroidogenic genes and serum steroid hormone levels, with a PCC threshold of 0.5 for the qRT-PCR and ELISA analyses. Cytoscape [51] was employed to illustrate the networks according to the PCC values.

### 3.10. In Vitro Stimulation of L. maculatus Testes with Cortisol

To investigate the effects of cortisol on steroidogenic gene expression, testis tissues of maturing 3-year-old *L. maculatus* were treated with cortisol in vitro. *L. maculatus* testis tissues were dissected and inoculated into 12-well plates, then cultured with cortisol at concentrations of 0 μg/mL (control) and 50 μg/mL, according to previous experiments. The tissue samples were collected at 12 h and 24 h after the incubation for RNA extraction. Subsequently, the changes in gene expression in the testes after cortisol in vitro culture were assessed by qRT-PCR.

## 4. Conclusions

In summary, HPG axis genes were comprehensively characterized in *L. maculatus*, with high conservation of copy numbers, phylogenies, structures and expression levels relative to other teleost species observed. The expression profiles indicated the key factors functioning essentially during the steroidogenesis of *L. maculatus* under salinity change. Specifically, the expression of genes involved in cortisol and 11-KT synthesis (*cyp17a*, *hsd3b1*, *cyp21a*, *cyp11c*, *hsd11b2*, and *hsd17b3*) showed generally upregulated expression in the head kidneys and testes, respectively. *Cyp11c* and *hsd11b2* were involved in the synthesis and metabolism of both cortisol and 11-KT, and their putative interaction may contribute to steroid hormone homeostasis in *L. maculatus* sexual development. All of these findings will provide significant insights into the steroidogenic enzyme cascade in teleost lineages and may contribute to reproductive manipulation in aquaculture.

## Data Availability

The transcriptome datasets used in this study can be found in the NCBI Sequence Read Archive (SRA), including brain-SRR7528887, stomach-SRR7528884, spleen-SRR7528888, liver-SRR7528886, gill-SRR7528883, testis-SRR7528885, and ovary-SRR2937376.

## Supplementary Materials

The following supporting information can be downloaded at: https://www.mdpi.com/article/10.3390/ijms231810905/s1.
