# Supplementary material for: Steroidogenic Effects of Salinity Change on the Hypothalamus–Pituitary–Gonad (HPG) Axis of Male Chinese Sea Bass (Lateolabrax maculatus)
Source: Int J Mol Sci. 2022 Sep 18;23(18):10905. doi: 10.3390/ijms231810905 (PMC9503316; doi:10.3390/ijms231810905)
Supplement: Supplementary file 1 [file ijms-23-10905-s001.zip › Figures S1-S3.pdf]

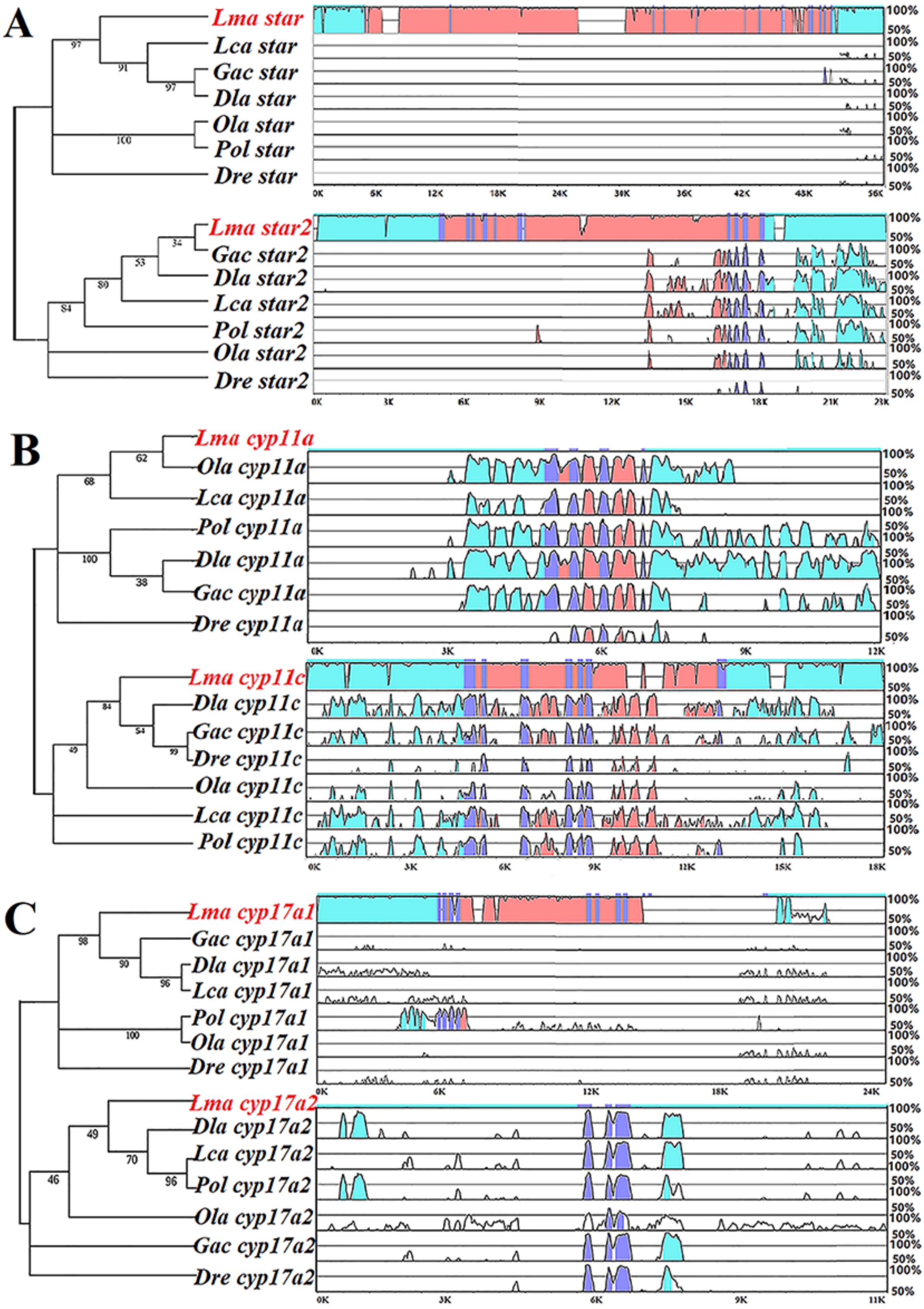

Figure S1: mVISTA analysis of (A) *star*/*star2*, (B) *cyp11a*/*cyp1c* and (C) *cyp17a1*/*cyp17a2*. Blue color represents intergenic regions, purple color represents exon region, orange color represents intron region. When the similarity of gene structure between *L. maculatus* and selected teleosts is higher than 50%, it will be displayed in color. The genes of the *L. maculatus* HPG axis are shown in red font, enlarged italic and bold.

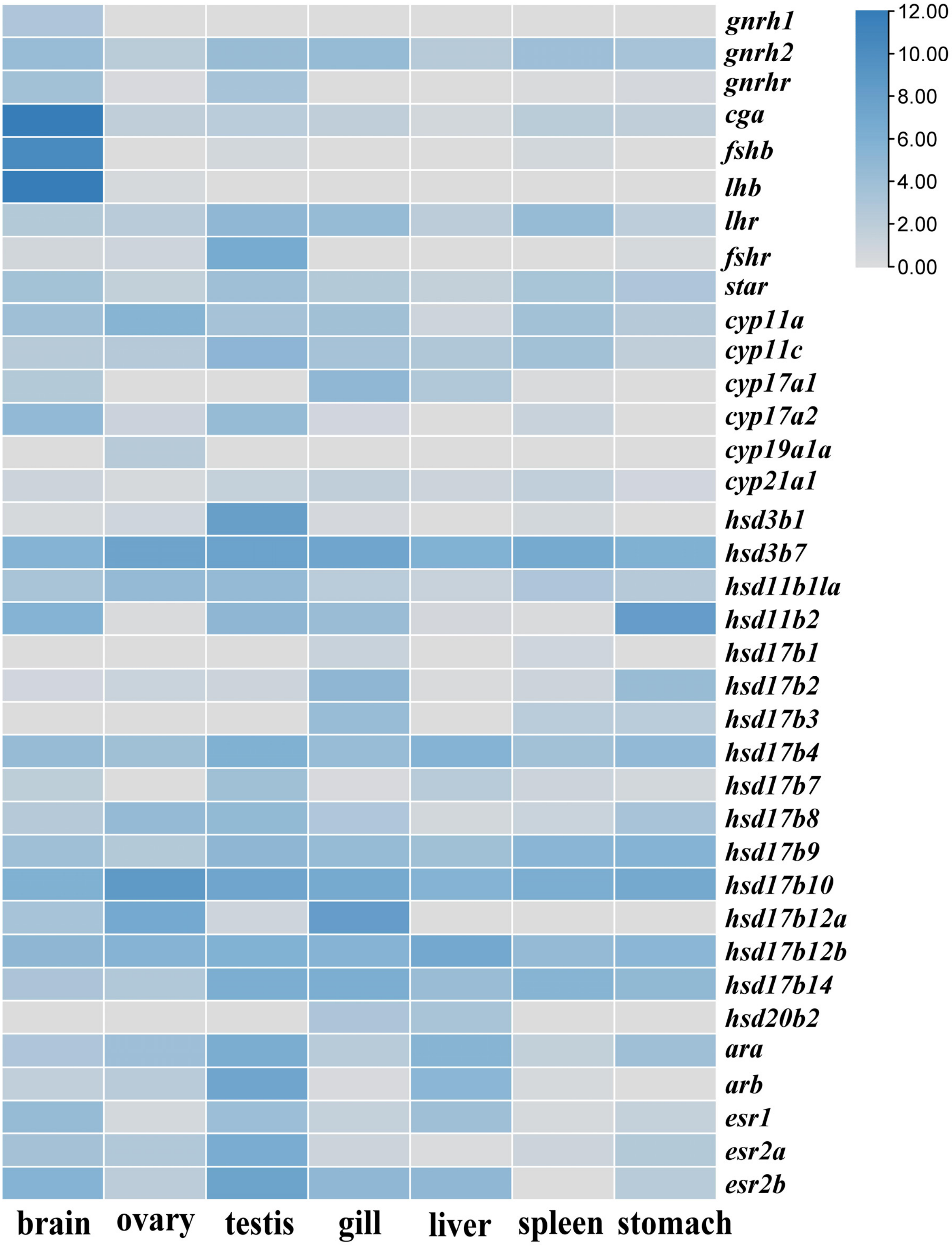

Figure S2: Expression profile of HPG axis genes in 7 adult tissues of *L. maculatus* with their $\log_2$(FPKM+1) values.

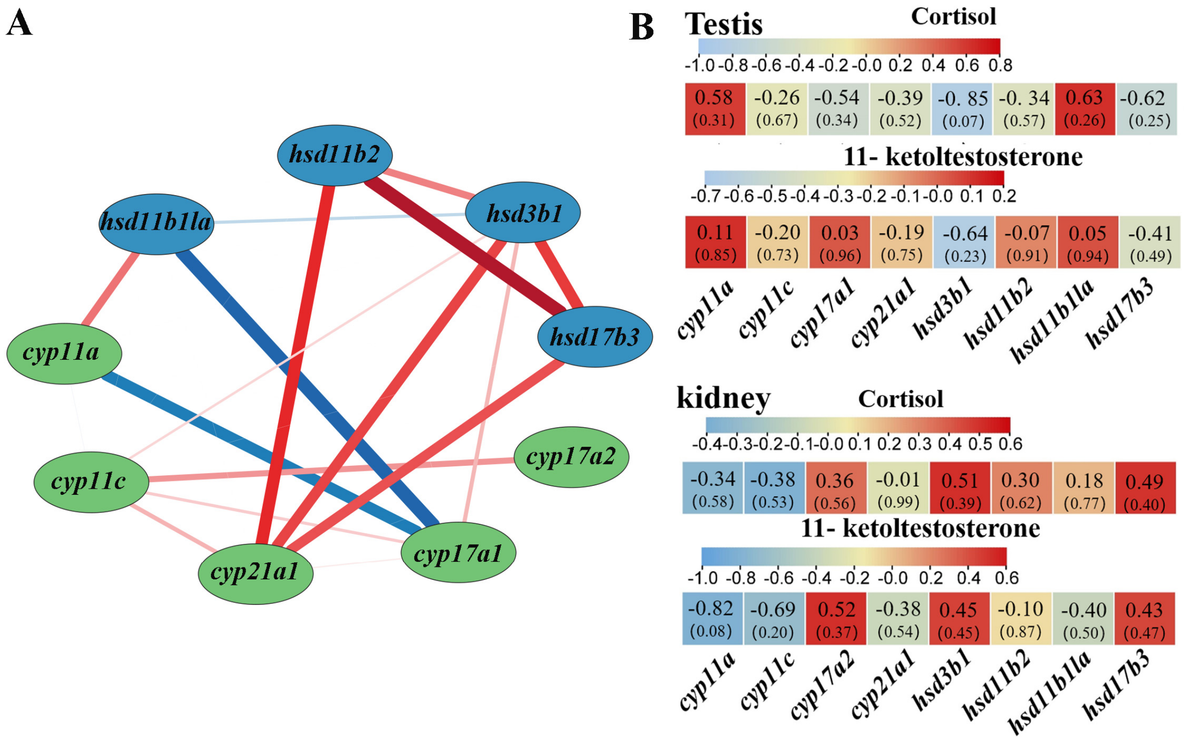

Figure S3: Pearson correlation of (A) steroid hormone metabolism pathway genes and (B) steroid hormones in response to salinity changes in *L. maculatus*. The thicker the line, the higher the correlation.
