# Supplementary material for: Steroidogenic Effects of Salinity Change on the Hypothalamus–Pituitary–Gonad (HPG) Axis of Male Chinese Sea Bass (Lateolabrax maculatus)
Source: Int J Mol Sci. 2022 Sep 18;23(18):10905. doi: 10.3390/ijms231810905 (PMC9503316; doi:10.3390/ijms231810905)
Supplement: Supplementary file 1 [file ijms-23-10905-s001.zip › Table S5.Primers.pdf]

Table S5. Primers used in this study.

**Primers**	**Sequence (5'-3')**	**Tm (℃)**	**Usage**
Lma-hsd3b1_RT_FW	GATTCGACTGCTGGACAAA	60	qRT-PCR
Lma_hsd3b1_RT_RW	GATGTGGAAGACGACTGATAC	60	qRT-PCR
Lma_hsd11b2_RT_FW	AAGCAGCTCTCAACCTCTTC	60	qRT-PCR
Lma_hsd11b2_RT_RW	CTGGACTGACCTGTCTTGTATG	60	qRT-PCR
Lma_hsd11b1la_RT_FW	AGGAGACCCTGAGTTCTCTATG	60	qRT-PCR
Lma_hsd11b1la_RT_RW	CGGTTGGAAAGACGAGGAAT	60	qRT-PCR
Lma_hsd17b3_RT_FW	CAGGCAGTTGCTCCATTT	60	qRT-PCR
Lma_hsd17b3_RT_RW	GTCTCCAGCTCTGAGGTATT	60	qRT-PCR
Lma_cyp11a1_RT_FW	CAGGATGACAGCACAGAATG	60	qRT-PCR
Lma_cyp11a1_RT_RW	CCCGAGATATTCTCCCAGTT	60	qRT-PCR
Lma_cyp11c1_RT_FW	GGAAGCGCAGTCTGGTTAT	60	qRT-PCR
Lma_cyp11c1_RT_RW	GATGAGGAGAAGAGGCCAATAC	60	qRT-PCR
Lma_cyp17a1_RT_FW	CTGCTTACTTCTGTCCTGTATG	60	qRT-PCR
Lma_cyp17a1_RT_RW	CCTGTTGCTCACCCATTT	60	qRT-PCR
Lma_cyp17a2_RT_FW	CTGTTGACCAGAGGAGGTAAAG	60	qRT-PCR
Lma_cyp17a2_RT_RW	CTCCAAACAGAGTGAAGGAGTT	60	qRT-PCR
Lma_cyp21a1_RT_FW	GTCCTGAGCGATATTCATGTT	60	qRT-PCR
Lma_cyp21a1_RT_RW	CTGTGCACAACTCCTCATAC	60	qRT-PCR
Lma_gnrh2_RT_FW	ACCCTCCCTCAACTTTACA	60	qRT-PCR
Lma_gnrh2_RT_RW	GTGGTGCTTGTTGTTGTTG	60	qRT-PCR
Lma_cga_RT_FW	TAGCACGCGATGACAATG	60	qRT-PCR
Lma_cga_RT_RW	ATGACGCCGTAACAATGG	60	qRT-PCR
Lma_gnrhr_RT_FW	GCCTCATGGACGGCATTTAT	60	qRT-PCR
Lma_gnrhr_RT_RW	TAGGCTGCACCAAGGTTAAAG	60	qRT-PCR
Lma_lhr_RT_FW	CAACAGCCTGAGGGTTATTGA	60	qRT-PCR
Lma_lhr_RT_RW	GCGCCAAGGAAGAGATAGTT	60	qRT-PCR
Lma_fshr_RT_FW	CCCTTTCTGTACGCCTTCTT	60	qRT-PCR
Lma_fshr_RT_RW	GGAACTCTCTGTCCGGTAAATC	60	qRT-PCR
Lma_18s_RT_FW	GGGTCCGAAGCGTTTACT	60	qRT-PCR
Lma_18s_RT_RW	TCACCTCTAGCGGCACAA	60	qRT-PCR
Lma_actb_RT_FW	CAACTGGGATGACATGGAGAAG	60	qRT-PCR
Lma_actb_RT_RW	TTGGCTTTGGGGTTCAGG	60	qRT-PCR
